# Retraction Note: Prevalence and associated factors of early initiation of breastfeeding among women delivered via Cesarean section in South Gondar zone hospitals Ethiopia, 2020

**DOI:** 10.1186/s40748-022-00145-x

**Published:** 2022-12-02

**Authors:** Bekalu Getnet, Alemu Degu, Fantahun Yenealem

**Affiliations:** 1 Department of Midwifery, College of Health Sciences, Debre Tabor University, P. Box: 272, Debre Tabor, Ethiopia; 2 Department of Midwifery, College of Medicine and Health Sciences, Bahir Dar University, Bahir Dar, Ethiopia

**Retraction Note: Matern Health Neonatol Perinatol 6, 6 (2020)**

**https://doi.org/10.1186/s40748-020-00121-3**

The Editor-in-Chief has retracted this article because the data presented derive from a different study [1] and were used without permission. None of the authors has responded to correspondence from the publisher about this retraction.
